# Using GANs with adaptive training data to search for new molecules

**DOI:** 10.1186/s13321-021-00494-3

**Published:** 2021-02-23

**Authors:** Andrew E. Blanchard, Christopher Stanley, Debsindhu Bhowmik

**Affiliations:** Computational Sciences and Engineering Division, Oak Ridge National Laboratory, Oak Ridge, TN 37830 USA

**Keywords:** Generative Adversarial Network, Drug discovery, Search

## Abstract

The process of drug discovery involves a search over the space of all possible chemical compounds. Generative Adversarial Networks (GANs) provide a valuable tool towards exploring chemical space and optimizing known compounds for a desired functionality. Standard approaches to training GANs, however, can result in mode collapse, in which the generator primarily produces samples closely related to a small subset of the training data. In contrast, the search for novel compounds necessitates exploration beyond the original data. Here, we present an approach to training GANs that promotes incremental exploration and limits the impacts of mode collapse using concepts from Genetic Algorithms. In our approach, valid samples from the generator are used to replace samples from the training data. We consider both random and guided selection along with recombination during replacement. By tracking the number of novel compounds produced during training, we show that updates to the training data drastically outperform the traditional approach, increasing potential applications for GANs in drug discovery.

## Introduction

From materials design to drug discovery, many scientific endeavors with significant practical applications can be viewed as a search over the space of all possible chemical compounds [1, 2]. Due to the high-dimensional nature of the search space, an exhaustive enumeration of possible candidates is not feasible [1]. To overcome this difficulty, traditional approaches in drug discovery have relied upon domain knowledge from physics and chemistry to construct synthesis rules to guide the search for new compounds. However, reliance on current knowledge to generate rules may unnecessarily limit the amount of chemical space explored [2].

In recent years, a data driven approach has emerged to empower searches over chemical space. Deep learning models have been constructed to learn lower dimensional representations of data to identify meaningful clusters and discover related compounds with a desired functionality [3–7]. Of particular interest to drug discovery, machine learning (ML) models have been incorporated into pipelines for iterative refinement of candidates. More specifically, generative models have been utilized as a key component for providing novel molecules for targeted experimental investigations [1, 2, 8].

Generative models in machine learning seek to recreate the distribution underlying a given set of data. After modeling the distribution, new samples can be drawn that extend the original data. One type of generative approach, known as a Generative Adversarial Network (GAN), has been widely used in many applications from image generation to drug discovery [9–12]. Recent studies have utilized GANs to search the space of possible molecules for drug design, developing models that can generate compounds with a desired feature set [11, 12].

Although generative models (and GANs) have many advantages for finding new molecules, a key limitation is the propensity for mode collapse [8, 13]. In mode collapse, the model distribution collapses to cover only a few samples from the training data. Beyond mode collapse, it is intuitively expected that a given generative model will be limited by the training data used (i.e. there is no standard way to guide the generative model in areas of parameter space that it has never encountered in training). This limitation hinders the use of GANs in search applications such as drug discovery. To overcome mode collapse, several approaches have been investigated including updating the loss function to promote diversity [12, 14, 15]. However, these approaches rely on comparisons to a fixed training data set, which continues to hinder search applications.

Here, we build upon recent work utilizing GANs for small molecule discovery [11] by introducing a new approach for training. Our approach enables augmented search through incremental updating of the training data set using ideas from Genetic Algorithms [16]. Novel and valid molecules that are generated by the model are stored during a training interval. Then, the training data is updated through a replacement strategy, which can be guided or random. Training resumes and the process is repeated. Our results show that this approach can alleviate the decrease in new molecules generated that occurs for a standard GAN during training. Furthermore, we utilize recombination between generated molecules and the training data to increase new molecule discovery. Introducing replacement and recombination into the training process empowers the use of GANs for broader searches in drug discovery.

## Results and discussion

To improve the search capabilities of GANs, we updated the training process to include concepts from Evolutionary (e.g. Genetic) Algorithms [16]. For a typical Genetic Algorithm, a parent population is used to generate a child population through mutation and recombination. The parent population is then updated (i.e. replaced) using selection based on specified fitness criteria. For our purposes, the training data is the population under consideration. The generator from the GAN produces candidates for the child population over multiple training epochs, and recombination occurs between the new candidates and the parent generation. Through replacement, the training data adapts to better reflect new areas explored by the generator.

As a first step in using adaptive training data for GANs, we consider replacement without recombination on a training set from QM9 [17]. In this case, we have three different types of training: control, random, drug. For control, the training data is held fixed while the GAN is trained. For random, the training data is updated by the generated molecules. For drug, the training data is updated only by generated molecules that outperform the current samples on quantitative estimation of drug-likeness score (i.e. drug-likeness) [18, 19]

**Fig. 1:**
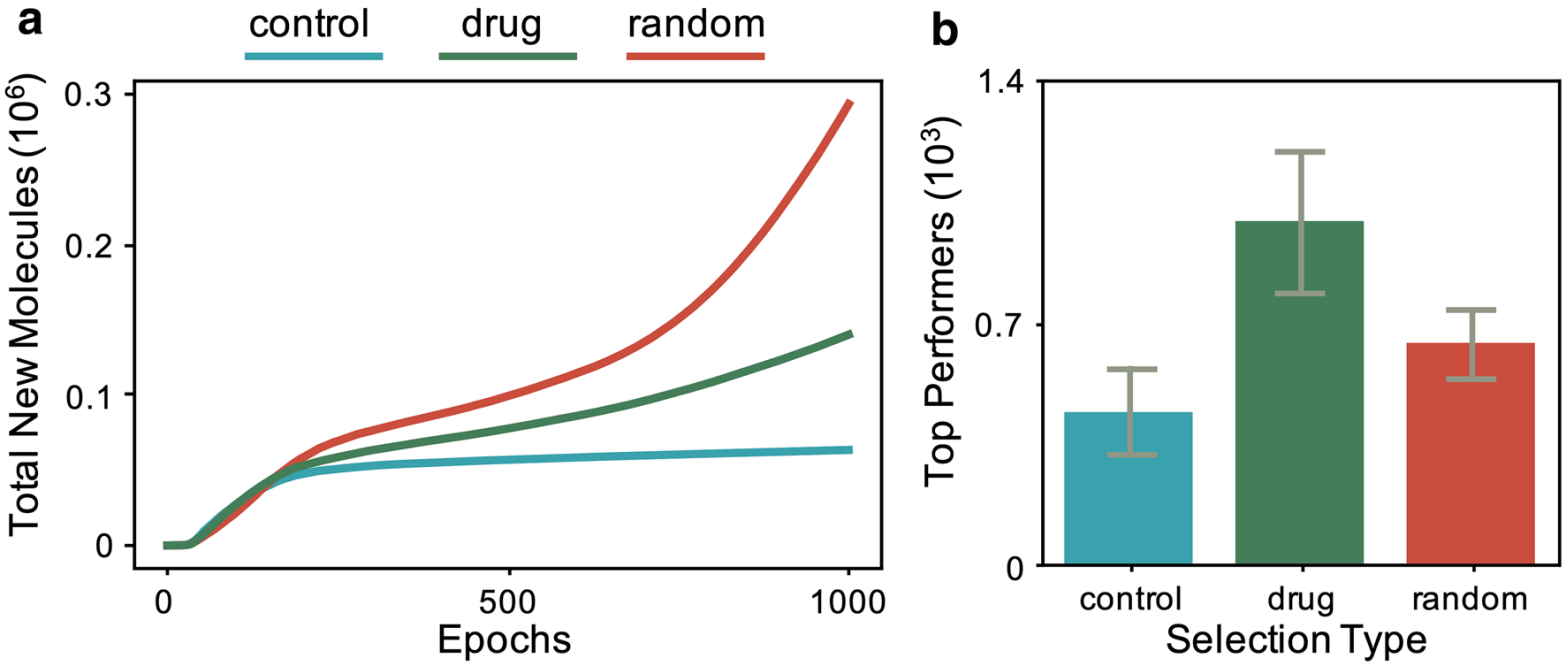
New molecules produced for different replacement strategies. For control (blue), the training data is fixed. For random (red), molecules from the generator randomly replace molecules in the training data. For drug (green), molecules from the generator only replace training samples if they have a higher drug-likeness score. **a** As training progresses, control stops producing a substantial number of new molecules, but random and drug replacement strategies continue production. Plot shows average over three training runs for each selection type. **b** Although drug produces less overall new molecules than random, it generates more top performers. Plot shows average over three runs for each selection type with error bars showing one standard deviation

As shown in Fig. 1a, the control GAN produces new molecules during the initial stage of training, but quickly reaches a plateau. Intuitively this is expected, as the generator learns to mimic the training data, the number of novel molecules produced decreases. Alternatively, for random and drug replacement, the GAN continues to produce new molecules over the entire training period as the training data is updated.

Although the number of new molecules produced is an important metric for drug discovery, when optimizing for some feature (e.g. drug-likeness), the quality of the generated samples is also key. As shown in Fig. 1b, drug replacement is able to generate the most top performers even though it generates fewer new molecules than random. Here, we define top performers as having a drug-likeness score above a threshold of 0.6, corresponding to the approximate mean value of optimized molecules in previous work [11, 12]. Similar results are shown for additional metrics (i.e. synthesizability and solubility) in Additional file 1: Figures S1, S2. Notice that the metric-specific selection strategy generates the most top performers for each metric considered.

**Fig. 2:**
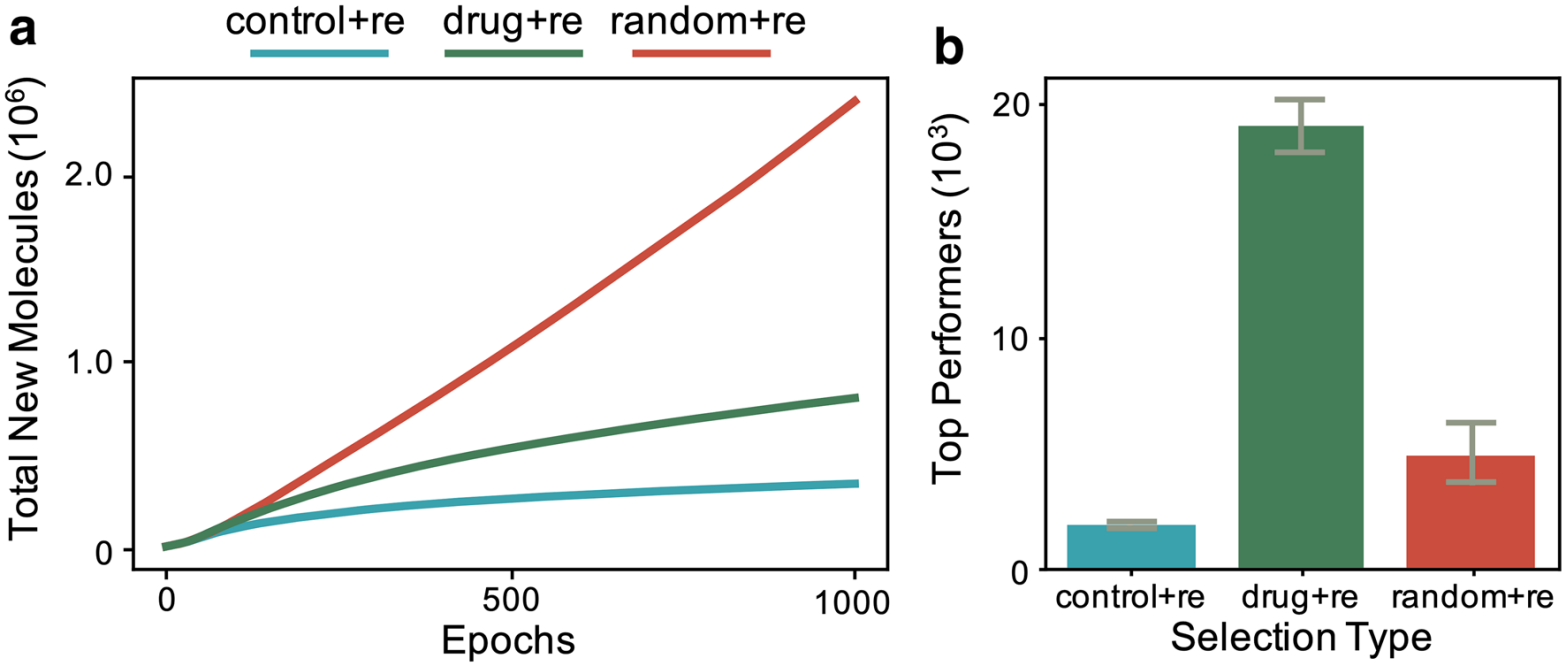
New molecules produced for different replacement strategies with recombination. For control+re (blue), the training data is fixed. For random+re (red), molecules from the generator randomly replace molecules in the training data. For drug+re (green), molecules from the generator only replace training samples if they have a higher drug-likeness score. **a** Similar to the case without recombination, random and drug replacement strategies outperform control as training progresses. Plot shows average over three training runs for each selection type. **b** Although drug+re produces less overall new molecules than random+re, it generates more top performers. Plot shows average over three runs for each selection type with error bars showing one standard deviation

In addition to selection/replacement, another common mechanism in Genetic Algorithms to introduce diversity into a population is recombination. We included recombination into our approach by taking half of the generated molecules and applying crossover with a sample from the current training data. As shown in Fig. 2a, the same hierarchy as the case without recombination is observed. Recombination, however, does increase the absolute number of new molecules produced drastically. The increase in new molecules also translates to many more high performers (Fig. 2b. Similar results are shown for additional metrics (i.e. synthesizability and solubility) in Additional file 1: Figures S3, S4.

**Fig. 3:**
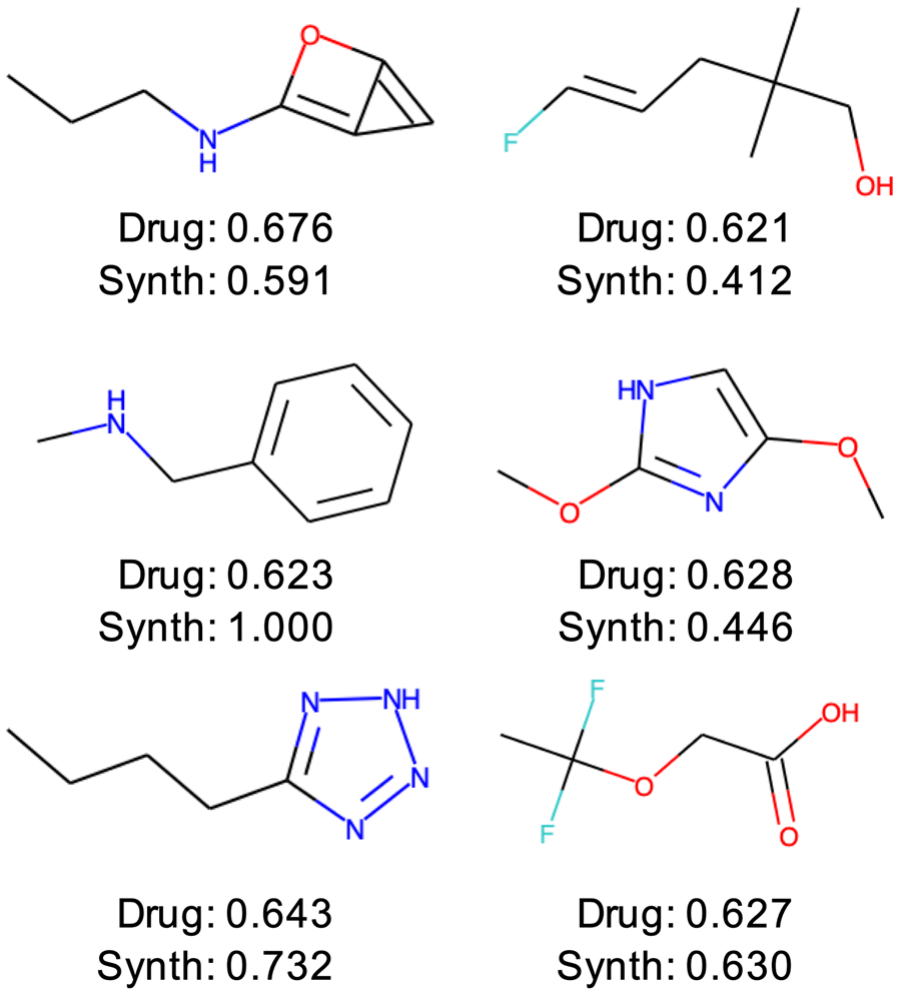
6 sample top performers produced by the GAN with drug replacement strategy and recombination. Quantitative estimation of drug-likeness score and synthesizability score computed using rdkit are shown

Beyond the bulk performance metrics shown in Fig. 2, Fig. 3 shows specific examples of top performers for the generator trained using drug replacement strategy and recombination. It is illustrative to consider the closest (as measured by Morgan fingerprints [20] and Tanimoto similarity) training set molecule for each example (Additional file 1: Figure S5). For most of the example top performers, only small rearrangements (e.g. changing an atom type or extending a chain) are necessary to provide a boost in the drug-likeness. The prevalence of small rearrangements in the generated molecules, however, is intuitively expected due to constraining the search space to molecules with 9 atoms or less.

**Fig. 4:**
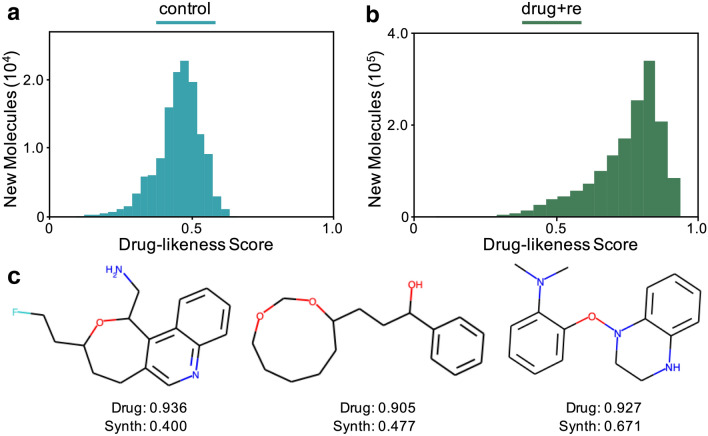
Training runs with molecules of 20 atoms or less. Results are shown for control (blue) and drug replacement with recombination (green). **a** Histogram showing number of new molecules produced in control run for different drug-likeness scores. **b** Histogram showing number of new molecules produced in our approach using updates to the training data for different drug-likeness scores. **c** A few sample new molecules from the drug replacement with recombination run

The success of guided training data replacement and recombination, as seen in the over 10x improvement over control (see Figs. 1, 2), motivated us to apply our approach to a more realistic data set for drug discovery. Therefore, we extended the training procedure to molecules with 20 or less atoms and added 10k molecules from the ZINC [21] rings data set (see "Methods" section). Our results (see Fig. 4) show that our approach again provides a drastic improvement over the traditional GAN for search. The total number of molecules produced increases over the control run (i.e. no replacement, no recombination) by an order of magnitude (${\sim }10^5$ to ${\sim }10^6$). Furthermore, the distribution of drug-likeness scores is altered drastically to favor high scoring compounds (Fig. 4a, b).

A sample of some of the top performers are shown in Figure 4c. The closest molecule from the training set for each top performer is shown in Figure S6. Unlike the data constrained to 9 atoms or less, the examples show substantial rearrangements of functional groups compared to the training set. Furthermore, the rearrangements result in a substantial boost in drug-likeness, which reflects the large shift in the histogram for produced molecules (Fig. 4a, b).

To more systematically understand the change in properties for molecules produced using selection and recombination compared to the traditional approach, we computed the distributions for additional metrics (Additional file 1: Figures S7–S11, Table S1). Additional file 1: Figure S7 shows the fraction of each molecule occupied by a given atom type (C, N, O, F). The molecules produced by the drug+re strategy show a shift towards higher C, F content and lower O, N content compared to the training data and control strategy. Additional file 1: Figure S8 shows the number of atoms, number of rings, and length of rings for the molecules. Again, the distributions for the drug+re strategy show a noticeable shift from the training data, with larger molecules, more rings, and larger rings.

An additional 3 metrics (number of rotatable bonds, polar surface area, and Crippen LogP [22]) are shown in Additional file 1: Figure S9. It is important to note that these metrics are commonly utilized to filter drug candidates [18, 23]. Both number of rotatable bonds and Crippen LogP show a substantial increase for the molecules from drug+re compared to the training data. The shifts for these metrics can be anticipated as they are both used to determine the drug-likeness score. Polar surface areas is also used in the drug-likeness score provided by rdkit but with a much smaller weight.

The shift of the drug+re distribution away from the original training data can also be quantified using fingerprint similarity. To determine the distance of produced molecules from the original data, we computed the Morgan fingerprints for each molecule in rdkit. We then found the closest molecule based on Tanimoto similarity (and corresponding distance). Additional file 1: Figure S10 shows the distributions of the minimum distance for both control and drug+re strategies. In agreement with the other metrics, molecules produced by the drug+re strategy on average have a larger minimum distance than the control molecules. Therefore, the drug+re strategy not only produces more molecules than control, it produces more distinct molecules relative to the training set.

As a final comparison, Additional file 1: Figure S11 shows the drug-likeness distributions across different selection and recombination strategies. The drug+re strategy shows a clear shift towards high scoring drug molecules compared to all other options. It is interesting to note that although recombination does provide a clear benefit in producing higher scoring molecules alone (i.e. compare control to control+re), updating the training data through selection generates a substantial shift in the probability density towards high performers.

The difficulty of mode collapse presents a major challenge to researchers using GANs for discovery. Previous attempts to prevent mode collapse have altered the loss function [14, 15], however, the issue has still remained in drug discovery efforts [11, 12]. Our approach, updating the training data, eliminated the plateau in new molecule discovery compared to the control case without any updates to the minimax loss function. Furthermore, recombination amplified the increase in new molecules for all replacement strategies. Together, these results suggest that replacement and recombination can drastically accelerate the use of GANs for drug discovery.

One limitation of the current approach is that a definition for valid generated samples must be given. In the current context, valid molecules are determined by the ability of rdkit to parse and create the proposed molecule. However, in other contexts, the definition of valid may not be so straightforward (e.g. what defines a valid image). In these cases, some scoring function must be introduced to determine replacement/validity. This highlights the importance of developing useful domain specific metrics for ML applications, including drug discovery [2].

Allowing updates to the training data provides much needed flexibility towards utilizing GANs in drug discovery. This can be seen in our search for drug-like compounds with 20 atoms or less. The initial training set only contained 10% of molecules with more than 9 atoms (the rest coming from QM9), however, through replacement and recombination, the search adapted to explore regions of parameter space with higher scores. The ability to adapt relieves some of the pressure in generating large data sets for each new desired task, as an incremental approach can be used.

Updates to the training data can be placed within a broader context of data augmentation for GANs. Recent work [24, 25] has explored ways to improve GAN training on images by augmenting labeled data while preserving the underlying target distribution. Data augmentation techniques are particularly relevant due to the inherent costs associated with manual labeling. In the context of drug discovery, our results show that search for novel compounds is broadened by allowing the GAN to explore regions of parameter space outside the original training set through incremental updates. The key tradeoff is that features of the original distribution may be lost as the training data shifts. The type of application and diversity of the training data can then be used to determine the costs and benefits associated with training data updates. In cases where labeled data is abundant and diverse, traditional approaches to training can be used. In cases with limited initial data, or limited initial data with desired characteristics, training updates can be used to improve search performance.

Our approach in updating the training data also has many connections to previous searches over chemical space using genetic algorithms [26–28]. For a genetic algorithm, hand-crafted rules are created for mutation (e.g. switch an atom type, delete an atom, add an atom) and recombination (e.g. swap functional groups between two molecules). Iterations of mutations and recombination are then performed on an initial population, with selection occurring to improve fitness in subsequent populations. In this context, the current work serves as a step towards automating the manual creation of rules for mutation. The generator network serves to produce candidate molecules based on the current training population, which is updated over time. Automating the process of recombination (in addition to mutation) is an interesting direction for future work.

Previous work has explored a possible way to incorporate genetic algorithms into GAN training [29]. More specifically, mutation and recombination were applied directly to the generator output in order to stabilize the training of the discriminator. This approach can be broadly categorized with other efforts to promote diversity during GAN training through data augmentation [24, 25] in contrast to our approach of updating the training data. It is important to note that improvements to model architecture and approaches to prevent overfitting, such as dropout, would not alleviate the need for updates to the training data in search. Approaches to prevent overfitting, by design, would enable the model to more fully reproduce the distribution underlying the training data, however, they would not promote exploration beyond the training data as needed in search applications.

Many of the advances in training GANs [14, 15] should be complementary to our approach. Here, we have utilized a relatively simple architecture, i.e. fully connected networks with a few layers for both generator and discriminator, and the standard GAN loss function. By adding replacement and recombination, however, large gains were seen in both the number and quality (i.e. drug score) of new molecules produced. The addition of more sophisticated networks (e.g. GCN [30]) to scale the current approach to larger molecules is an interesting direction for future investigations.

## Conclusions

Generative machine learning models, including GANs, are a powerful tool towards searching chemical space for desired functionalities. Here, we have presented a strategy for promoting search beyond the original training set using incremental updates to the data. Our approach builds upon the concepts of selection and recombination common in Genetic Algorithms and can be seen as a step towards automating the typically manual rules for mutation. Our results suggest that updates to the data enable a larger number of compounds to be explored, leading to an increase in high performing candidates compared to a fixed training set.

## Methods

### Data

The original training data used for all models was taken from QM9 [17], a subset of the GDB-17 chemical database [31], as reported in a previous study [11]. The data was downloaded from deepchem^1^ and then processed using rdkit [19], with any molecules that caused errors during sanitization removed. Only the first 100k (out of $\sim 133\hbox {k}$) compounds were then used in training.

To modify the training data to include larger molecules (i.e. up to 20 atoms), a subset of the ZINC [21] rings data set was used. Smiles data was downloaded from ZINC^2^ and filtered to include molecules with between 10 and 20 atoms that contain only C, N, and O. The first 10k molecules were then used to replace the first 10k entries from the original training data. The resulting training data had 100k molecules, with 90k from QM9 [17] and 10k from Zinc [21].

### Models

The GAN was implemented using pytorch [32], with both the discriminator and generator consisting of 4 fully connected layers. The generator received as input normally distributed random vectors with dimension 8. The output of the generator was an adjacency matrix with the off-diagonal elements specifying the bond order and the on-diagonal elements specifying the atom type. The discriminator received as input the one hot representation of the adjacency matrix and output a single real number. We utilized the standard GAN minimax loss for training [9]. The Adam optimizer[33] was used with a learning rate of $10^{-4}$ for all runs.

### Updates to training data

All models were trained in intervals of 5 epochs. During each epoch 10k samples were collected from the generator. Samples that were both novel and valid were aggregated over the 5 epochs. Then, a replacement strategy (random or drug) was applied to the original training data. For random replacement, current training samples were randomly selected and replaced. For drug replacement, current samples were sorted in ascending order of drug-likeness score. Updates were only made if the new sample had a greater score than the sample being replaced. Additional metrics (i.e. synthesizability and solubility) used the same update procedure as drug-likeness.

For both replacement strategies, we also considered recombination. In recombination, half of the 10k generator sample was combined with the current training data using crossover. In crossover, a sample is selected from the current training data and copied into a new adjacency matrix. Then, a random integer is uniformly sampled between 1 and the length of the adjacency matrix. The corresponding slice from the generated matrix (e.g. first 5 rows and columns) overwrites the same region of the copied matrix to produce a new candidate molecule. For the drug replacement strategy, samples were drawn from the training set weighted by drug score. The weights were determined by taking the softmax of the current metric scores for the training data. Replacement then proceeded as previously stated. Additional metrics (i.e. synthesizability and solubility) used the same update procedure as drug-likeness.

### Metrics

To track the performance of each GAN during training, we relied upon two key metrics: the number of new molecules generated and the quantitative estimation of drug-likeness score [11, 18, 19]. Determining that a molecule is new was accomplished by comparing the canonical smiles representation of the compound with the full training set and any molecules produced up to that point. Generation of a canonical smiles string for a given molecule was performed using rdkit [19]. To show that the results are not unique to drug-likeness, we also included runs for selection of synthesizability and solubility. The metrics calculations were performed as previously reported [11, 12, 18, 34].

Additional metrics for analysis as shown in Additional file 1: Figures S7-S11 were all computed using built-in functionality from rdkit [19]. Similarity (and corresponding distance) between molecules was computed by generating Morgan fingerprints [20] and using Tanimoto similarity as preformed in a previous study [11].

## Supplementary Information

**Additional file 1: Supporting Information.**: Figures S1–S11 and Table S1.

## Data Availability

The training data used for this study can be downloaded as described in "Methods" section (Data subsection).

## Footnotes

1 http://deepchem.io.s3-website-us-west-1.amazonaws.com/datasets/gdb9.tar.gz.

2 https://zinc.docking.org/rings.smi?count=all.
