# Supplementary material for: Using GANs with adaptive training data to search for new molecules
Source: J Cheminform. 2021 Feb 23;13:14. doi: 10.1186/s13321-021-00494-3 (PMC7901067; doi:10.1186/s13321-021-00494-3)
Supplement: Supplementary file 1 — Additional file 1: Supporting Information.: Figures S1–S11 and Table S1. [file 13321_2021_494_MOESM1_ESM.pdf]

Supporting Information: Using GANs with Adaptive Training Data to Search for New Molecules

Andrew E. Blanchard,* Christopher Stanley,* and Debsindhu Bhowmik*

Computational Sciences and Engineering Division, Oak Ridge National Laboratory, Oak Ridge, TN 37830 USA

E-mail: blanchardae@ornl.gov; stanleycb@ornl.gov; bhowmikd@ornl.gov

This manuscript has been authored by UT-Battelle LLC under Contract No. DE-AC05-00OR22725 with the US Department of Energy (DOE). The US government retains and the publisher, by accepting the article for publication, acknowledges that the US government retains a nonexclusive, paid-up, irrevocable, worldwide license to publish or reproduce the published form of the manuscript, or allow others to do so, for US government purposes. DOE will provide public access to these results of federally sponsored research in accordance with the DOE Public Access Plan (http://energy.gov/downloads/doe-public-access-plan).

Figure

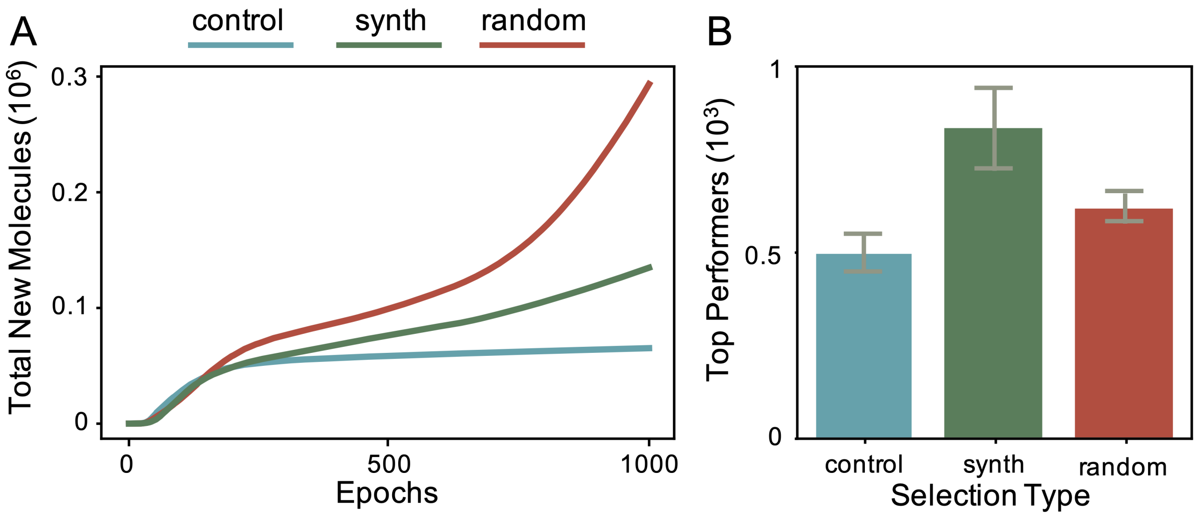

Figure S1: New molecules produced for different replacement strategies. For control (blue), the training data is fixed. For random (red), molecules from the generator randomly replace molecules in the training data. For synth (green), molecules from the generator only replace training samples if they have a higher synthesizability score. **A.** As training progresses, control stops producing a substantial number of new molecules, but random and synth replacement strategies continue production. Plot shows average over three training runs for each selection type. **B.** Although synth produces less overall new molecules than random, it generates more top performers. Here, we define top performers as having a synthesizability score above a threshold of 0.75, corresponding to the approximate mean value of optimized molecules in previous work.[1,2] Plot shows average over three runs for each selection type with error bars showing one standard deviation.

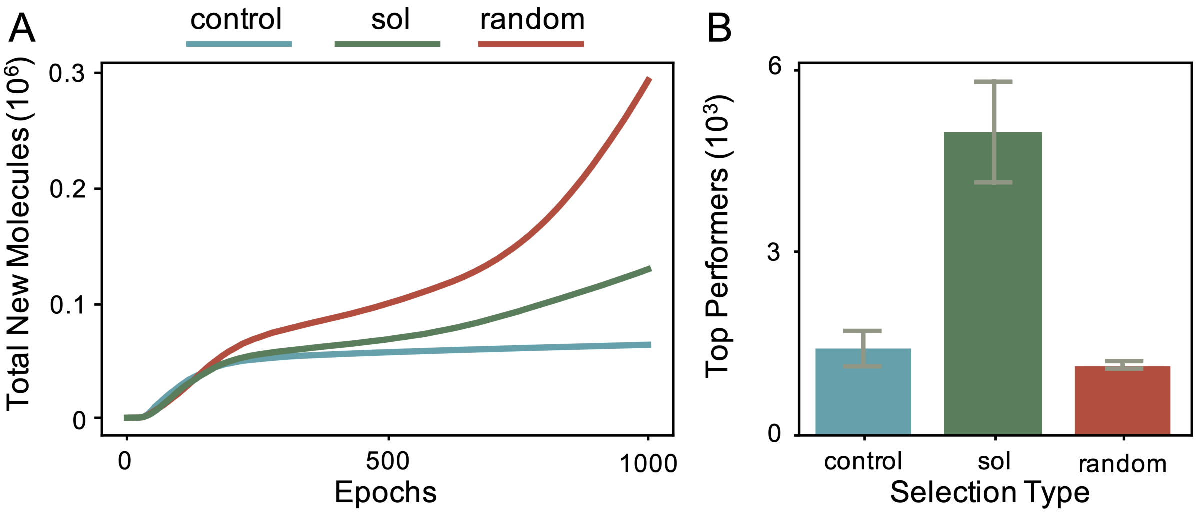

Figure S2: New molecules produced for different replacement strategies. For control (blue), the training data is fixed. For random (red), molecules from the generator randomly replace molecules in the training data. For sol (green), molecules from the generator only replace training samples if they have a higher solubility score. **A.** As training progresses, control stops producing a substantial number of new molecules, but random and sol replacement strategies continue production. Plot shows average over three training runs for each selection type. **B.** Although sol produces less overall new molecules than random, it generates more top performers. Here, we define top performers as having a solubility score above a threshold of 0.54, corresponding to the approximate mean value of optimized molecules in previous work[1,2]. Plot shows average over three runs for each selection type with error bars showing one standard deviation.

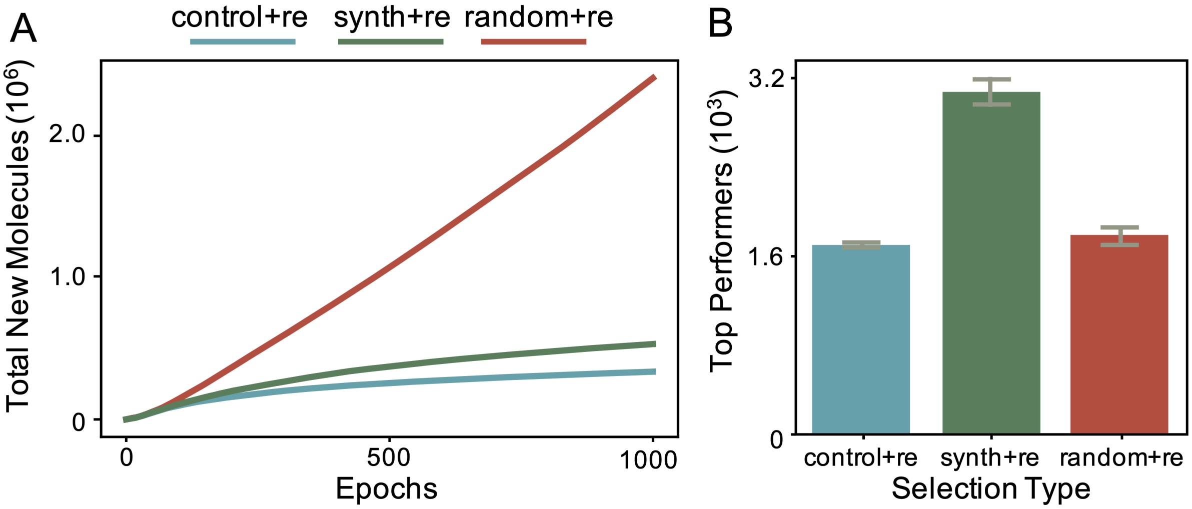

Figure S3: New molecules produced for different replacement strategies with recombination. For control+re (blue), the training data is fixed. For random+re (red), molecules from the generator randomly replace molecules in the training data. For synth+re (green), molecules from the generator only replace training samples if they have a higher synthesizability score. **A.** Similar to the case without recombination, random and synth replacement strategies outperform control as training progresses. Plot shows average over three training runs for each selection type. **B.** Although synth+re produces less overall new molecules than random+re, it generates more top performers. Here, we define top performers as having a synthesizability score above a threshold of 0.75, corresponding to the approximate mean value of optimized molecules in previous work.[1,2] Plot shows average over three runs for each selection type with error bars showing one standard deviation.

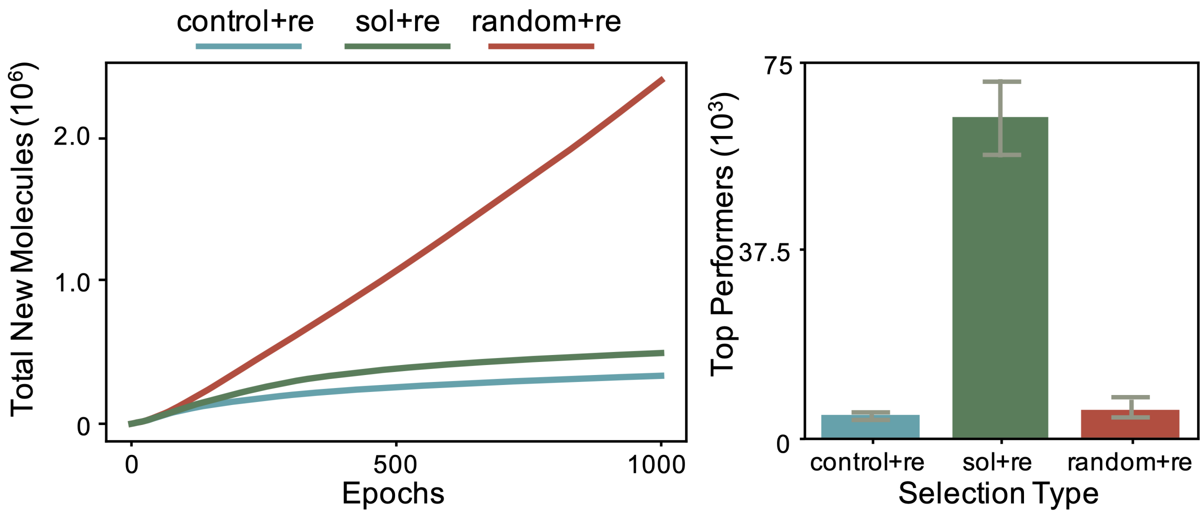

Figure S4: New molecules produced for different replacement strategies with recombination. For control+re (blue), the training data is fixed. For random+re (red), molecules from the generator randomly replace molecules in the training data. For sol+re (green), molecules from the generator only replace training samples if they have a higher solubility score. **A.** Similar to the case without recombination, random and sol replacement strategies outperform control as training progresses. Plot shows average over three training runs for each selection type. **B.** Although sol+re produces less overall new molecules than random+re, it generates more top performers. Here, we define top performers as having a solubility score above a threshold of 0.54, corresponding to the approximate mean value of optimized molecules in previous work[1,2]. Plot shows average over three runs for each selection type with error bars showing one standard deviation.

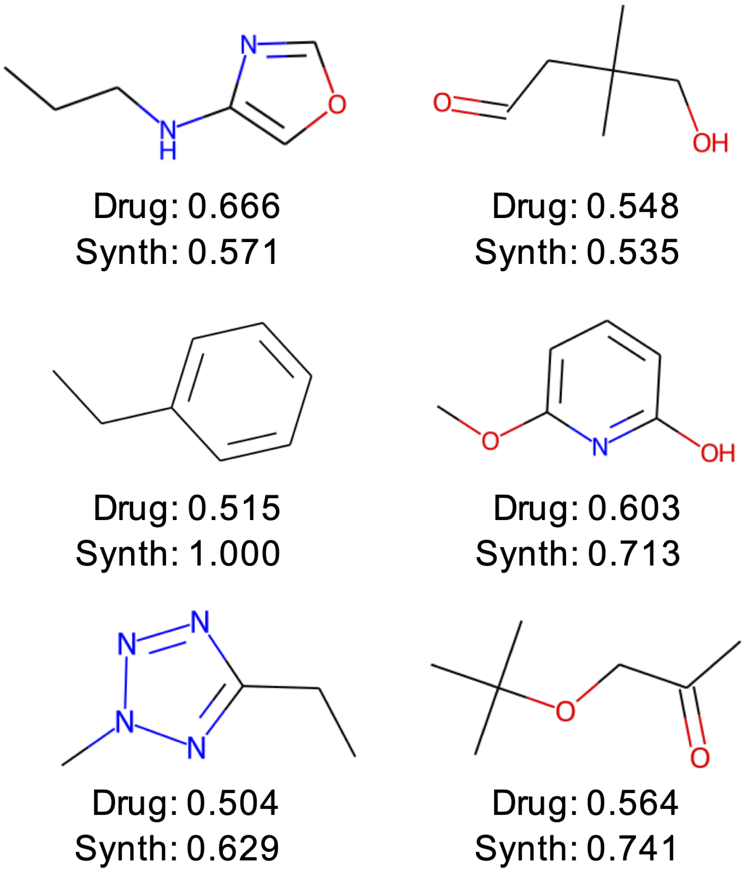

Figure S5: Closest (as measured by fingerprint similarity shown in [2]) molecules in the original training data to the 6 sample top performers shown in main text Figure 3. Quantitative estimation of drug-likeness and synthesizability are shown below each molecule.

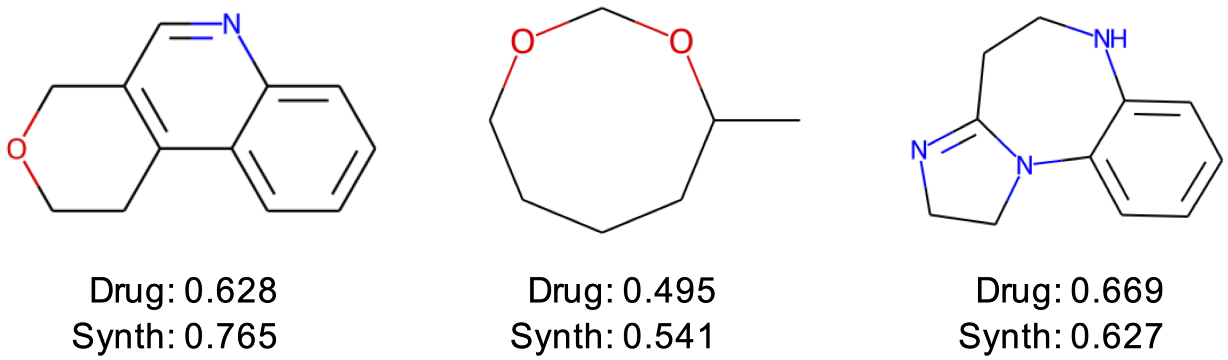

Figure S6: Closest (as measured by fingerprint similarity shown in[2]) molecules in the original training data to the 3 sample top performers shown in main text Figure 4C. Quantitative estimation of drug-likeness and synthesizability are shown below each molecule.

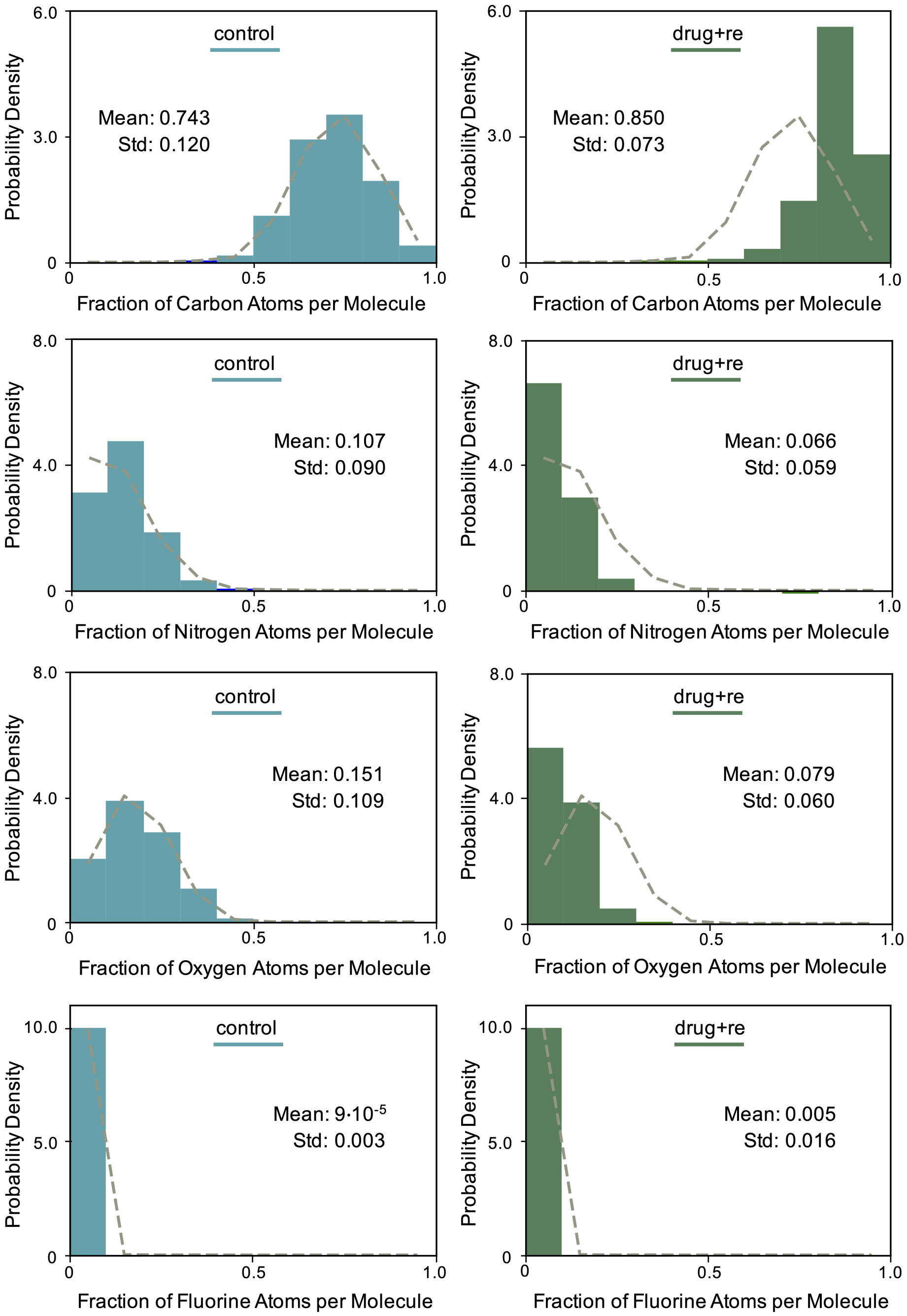

Figure S7: Histograms showing probability density for fraction of atom types in the molecules produced during training. The dashed line shows the histogram for the original training data.

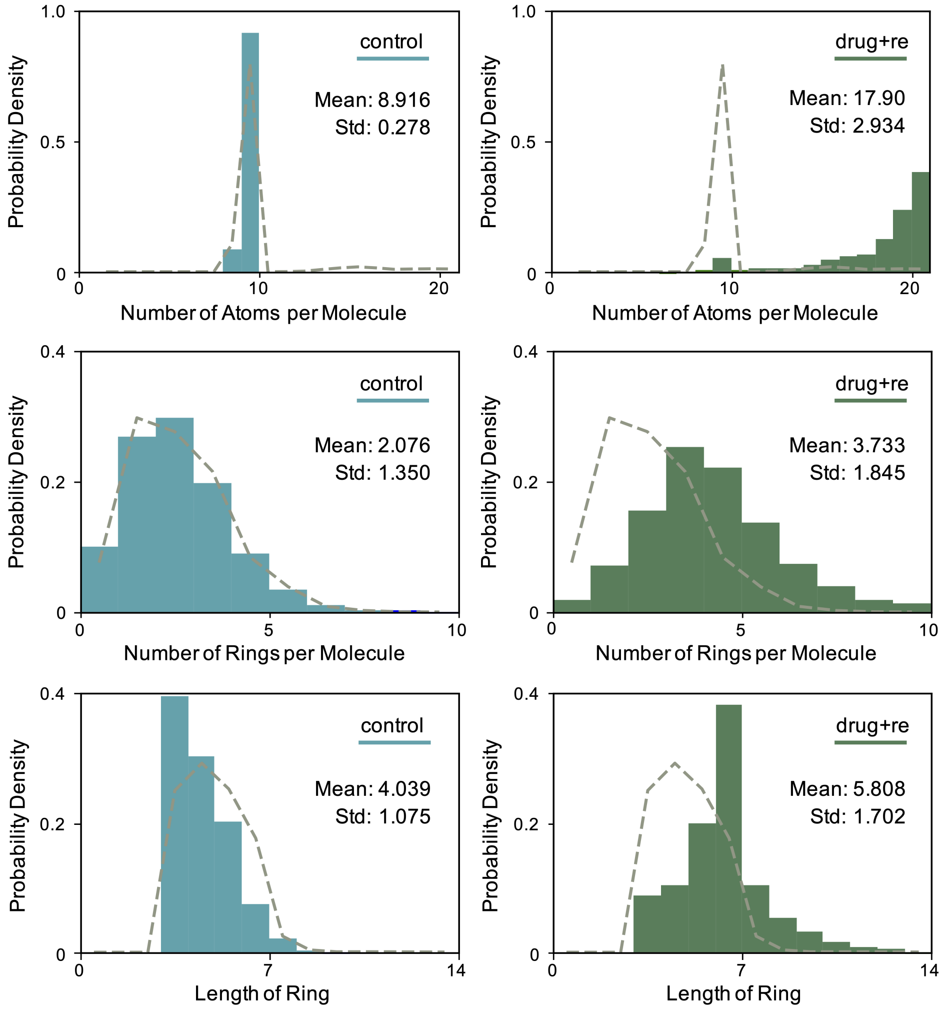

Figure S8: Histograms showing probability density for number of atoms per molecule, rings per molecule, and length of rings. The dashed line shows the histogram for the original training data.

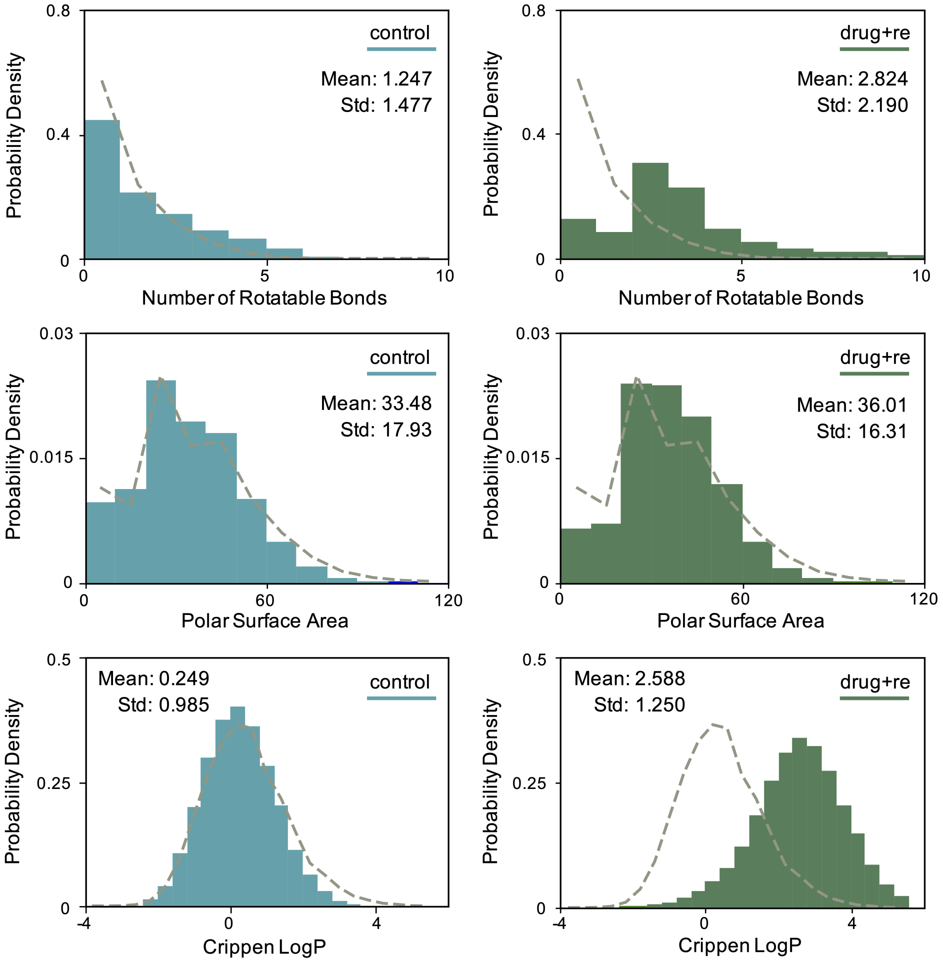

Figure S9: Histograms showing probability density for number of rotatable bonds, polar surface area, and Crippen LogP. The dashed line shows the histogram for the original training data.

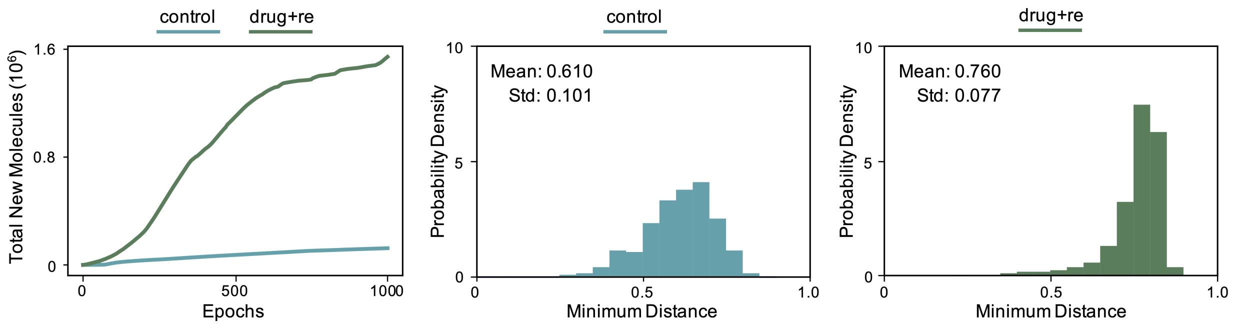

Figure S10: Total molecules produced by control and drug+re strategies. In addition to producing more molecules, the drug+re strategy produces molecules that are less similar to the training set compared to the control strategy. Here, the minimum Tanimoto distance (as computed in rdkit for Morgan fingerprints) is calculated for each produced molecule in reference to the training set.

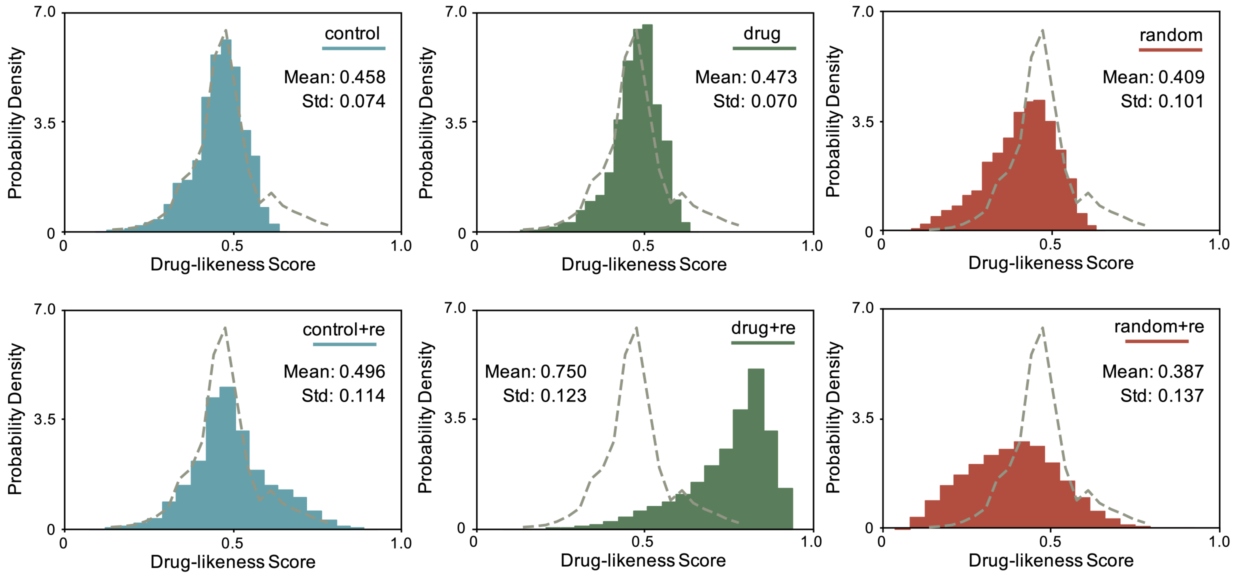

Figure S11: Histograms showing probability density for molecules produced during training with different selection strategies. The dashed line shows the histogram for the original training data.

Tables

Table S1: Means and standard deviations for metrics in original training data.

Metric	Mean	Std
Drug-likeness	0.476	0.094
Number of Atoms	9.618	2.337
Fraction C	0.749	0.120
Fraction N	0.098	0.010
Fraction O	0.153	0.099
Fraction F	0.001	0.012
Ring Count	2.102	1.311
Ring Length	4.468	1.214
Rotatable Bonds	0.713	1.016
Polar Surface Area	34.77	19.62
Crippen LogP	0.443	1.138

References

(1) Guimaraes, G. L.; Sanchez-Lengeling, B.; Outeiral, C.; Farias, P. L. C.; Aspuru-Guzik, A. Objective-Reinforced Generative Adversarial Networks (ORGAN) for Sequence Generation Models. 2017; `http://arxiv.org/abs/1705.10843`.

(2) De Cao, N.; Kipf, T. MolGAN: An implicit generative model for small molecular graphs. *ICML 2018 workshop on Theoretical Foundations and Applications of Deep Generative Models* **2018**,
